# Role of Dopaminergic Receptors in Glaucomatous Disease Modulation

**DOI:** 10.1155/2013/193048

**Published:** 2013-06-26

**Authors:** Nicola Pescosolido, Francesco Parisi, Paola Russo, Giuseppe Buomprisco, Marcella Nebbioso

**Affiliations:** ^1^Department of Cardiovascular, Respiratory, Nephrologic, Anesthesiologic and Geriatric Sciences, Sapienza University of Rome, Piazzale Aldo Moro 5, 00161 Rome, Italy; ^2^Department of Sense Organs, Centre of Ocular Electrophysiology, Sapienza University of Rome, Piazzale Aldo Moro 5, 00161 Rome, Italy

## Abstract

Both studies on animals and humans suggest the presence of dopamine (DA) receptors in the anterior segment of the eye. Their role in the dynamics of intraocular pressure (IOP) is not yet clear. DA_2_ and DA_3_ receptors are mainly located on postganglionic sympathetic nerve endings. Their stimulation reduces the release of norepinephrine and suppresses the production of aqueous humor. DA_1_ receptors seem to be more expressed by the ciliary body and the outflow pathway of aqueous humor. The administration of DA_1_-selective agonists stimulates the production of aqueous humor, increasing IOP, whereas DA_2_- and DA_3_-selective agonists could reduce IOP and, therefore, the risk to develop a glaucoma (GL). GL is a broad spectrum of eye diseases which have in common the damage to the optic nerve and the progressive loss of the visual field. Further studies are desirable to clarify the role of the dopaminergic system and the usefulness of DA_2_ and DA_3_ agonists in reducing IOP.

## 1. Introduction

Biological basis of glaucoma (GL) are not yet fully understood, and the factors involved in its progression are not yet fully identified. The most important risk factor is high intraocular pressure (IOP), due to the reduction in the outflow of aqueous humor through the iridocorneal angle [1, 2]. Other risk factors are aging (in elderly the prevalence of the disease is higher), family history, African-Caribbean race, high myopia (>4D), thinner cornea, lens pseudoexfoliation, low blood pressure (diastolic one in particular), and local and systemic vascular risk factors (optic disc hemorrhage or atrophy, vasospasm, etc.) [1–4].

Physiologically, IOP is regulated by a proper balance between secretion and drainage of aqueous humor. This is produced by ciliary body's epithelium into the posterior chamber from which passively diffuses into the anterior one through pupil [3–5]. Once in the anterior chamber, about 80% of aqueous humor outflows through the trabecular meshwork and then in a small vessel called Schlemm's canal. Finally, through aqueous veins, it comes out from ocular bulb by episcleral veins and reaches the bloodstream. The remaining 20% of aqueous humor flows, independently from IOP, through the uveoscleral outflow or along nerves and small ocular vessels [4–7]. 

High IOP is one of the most important risk factors involved in GL and is the only one against whom actual therapies are really effective. Epidemiological studies showed that the risk of GL increases by 12% for each 1 mmHg increase of IOP [1, 2, 8]. To date, GL is the second leading cause of blindness in the world [8]. GL can be congenital or acquired. The further classification in primary open-angle glaucoma (POAG) and primary angle-closure glaucoma (PACG) concerns the mechanisms that hinders aqueous humor outflow. In primary GL, the increase of IOP is not associated with other ocular disorders, while in secondary GL, a recognizable factor, ocular or nonocular one, alters the outflow of aqueous humor. In western countries, POAG is more common than PACG [1, 2, 8]. 

Molecular events responsible for GL are still for the most part unknown. Several molecules are able to regulate IOP: adrenergic, cholinergic, serotonergic, and dopaminergic systems are all involved. 

Dopamine (DA) is a simple organic chemical in the catecholamine family released from postganglionic nerve fibers of the superior cervical dopaminergic ganglion in the aqueous humor [9–12] and exerts its action by binding to 5 different types of DA receptors (DA_1–5_) which belong to the superclass of G-proteins coupled receptors. 

**Figure 1:**
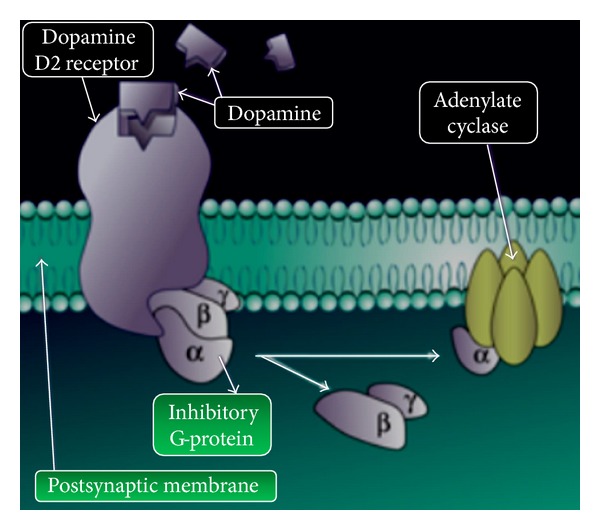
Structures of dopamine receptors (DA_2_/DA_3_) and G-protein coupled to receptors.

DA receptors are grouped in two distinct families: DA_1_-like receptors (which include DA_1_ and DA_5_ receptors) and DA_2_-like receptors, like DA_2_, DA_3_, and DA_4_ [11–14]. The main difference between those families of receptors concerns the action of G-proteins: DA_1_-like receptor has a Gs-protein whose activation results in an increase of cyclic AMP (cAMP) mediated by adenylate cyclase [9, 10, 12]. DA_2_-like receptors are coupled to a Gi-protein which determines, instead, the inhibition of adenylate cyclase and, consequentially, a decrease of cAMP (Figure 1).

The aim of this review is to investigate, specifically, the dopaminergic influence on IOP to evaluate the role of new hypotensive drugs. 

## 2. Studies on Animals 

Early studies of the role of DA in GL on animal models led to contrasting results. Shannon et al. [15] showed that DA, administered topically, increases IOP in rabbit eyes, and haloperidol (DA inverse agonist) can contrast that action. Green and Elijah [16] noticed, by contrast, that IOP decreased during parenteral administration of DA, indicating that a different route of administration of DA produces different effects on IOP. In 1984, Potter et al. [17] studied the sequel of topical administration of DA and its methylated analogs: N-methyl-dopamine (NMDA), N,N-dimethyl dopamine (DMDA), and N,N-Di-n-propyl dopamine (DPDA) in eyes of healthy rabbits and of sympathectomized ones. Thus, in normal rabbits, DA, DMDA, and NMDA induced an increase of IOP unilaterally, while DPDA induced a bilateral hypotension after unilateral topical administration.

Furthermore, in sympathectomized rabbits they noted the following results:

1. the administration of NMDA and DMDA produced an exaggerated ocular hypertension, followed by hypotension;
2. the hypotensive effects of DPDA registered in normal rabbits eyes were absent in sympathectomized ones.

In summary, they assessed that DA and its analogues produce two different effects:

- a direct and postjunctional effect, mediated by DA agonists that bind to DA_1_, *α*, and *β* receptors;
- an indirect and prejunctional (or “neuronal”) effect, mediated by DA agonists that bind *α*_2_ and DA_2_ receptors.

Green et al. [9, 16], studying rabbit's ciliary epithelium in vitro, observed that DA increases passive permeability and active secretion of aqueous humor. Both effects could be inhibited by *α* and *β* antagonists, phentolamine, and sotalol, respectively, but not by a relatively selective DA antagonist (butaclamol) [18]. The secretory function of DA was subsequently confirmed by studies that identified, on nonpigmented epithelium of the ciliary body of animals and humans, the phosphoprotein 3′–5′ monophosphate (DARPP-32) which regulates adenosine and DA systems [19]. 

Potter et al., however, showed that topical administration of some ergoline (lergotrile, pergolide) and an ergopeptide (bromocriptine), both in rabbits and monkeys, decreased IOP by acting on DA_2_ receptors [17, 20, 21]. The action of DA_2_ receptors was further confirmed by a subsequent study by Potter [12]. He noted a decrease hypotensive effect of ergoline in eyes of rabbits pretreated with domperidone (a DA_2_ antagonist) and surgical sympathectomy.

In summary:

1. the decrease of IOP mediated by ergot derivatives is partly dependent to sympathetic neuronal activity;
2. probably DA_2_ receptors are expressed in the sympathetic ganglion and/or sympathetic postganglionic nerveendings;
3. ocular hypotension is just partially due to the inhibition of formation of aqueous humor. To further confirm that indirect effects are responsible for lowering IOP, Savolainen et al. [22] reported that selective DA_2_/*α*_2_ agonist like CHF1035 and its metabolite CHF1024, if instilled in rabbit's eyes, have similar effects to brimonidine in lowering IOP, but CHF1035 has a long lasting action too.

These data suggest the presence of DA receptors in the anterior segment, but in order to better understand and investigate their role, it was important to precisely identify where the structures were present. Many biochemical, immunohistochemical and functional studies have been performed on animal tissue samples to find DA_1_ receptors localization, but not DA_2_ ones. DA_1_ receptors are present on ciliary body epithelium of bovine [11, 19] and on ciliary processes epithelium of rabbit [9, 15, 18] confirming that their stimulation causes from one side an increased production of aqueous humor and, on the other hand, an altered outflow of it affecting the tone of ciliary muscle. DA_2_ receptors main localization seems to be the postganglionic presynaptic nerve ending [22].

In addition to the previously mentioned receptors, Chu et al. showed the involvement of a specific DA receptor, the DA_3_, responsible for IOP hypotonization in an animal model of rabbit [23]. Recently, some authors studied the hypotensive effect of 7-hydroxy-2-dipropilaminotetralina (7OH-DPAT), the main agonist of DA_3_ receptor on rabbit eyes [23], showing that

1. IOP lowering effect of 7-OH-DPAT was due in part to the reduction of the production of aqueous humor;
2. the nervous site of action of 7-OH-DPAT has been identified immunohistochemically as the DA_3_ receptor of ciliary body and has been associated with the decrease of hypotensive effects in rabbits deprived of sympathetic nerve endings;
3. the inhibition of the hypotensive effect of 7-OH-DPAT by DA_3_ receptor antagonists (U99194A and UH232) and the deprivation of postganglionic sympathetic endings, together with the immunohistochemical data, showed that the primary site of action of 7-OH-DPAT is located in the presynaptic postganglionic nerve endings of the ciliary body of rabbit eyes.

A similar study, conducted on mice, was performed by Bucolo et al. [14]. The authors observed that topical application of 7-OH-DPAT, *α* DA_3_ preferring receptor against, significantly decreased in wild-type (WT) mice both in an ocular normotensive group and an ocular hypertensive steroid-induced group. Instead, there were no changes of IOP in DA_3_ receptor knockout mice. Moreover, genetic analysis conducted on ocular tissues of WT mice with PCR showed several genes encoding for all DA receptors but the absence of the receptor gene in DA_3_ KO mice [14].

## 3. Studies on Humans 

The expression of DA_2_ receptors in the anterior segment of the eye in humans has been confirmed by functional data, but there are no studies which revealed their localization. 

Unlike DA_2_ receptors, DA_1_ have also been identified in human eyes [19] with biochemical and autoradiographic techniques using [3]-SCH-23390, a selective antagonist of DA_1_ receptor. DA_1_ receptors were present on uveoscleral tissue, trabecular meshwork, and ciliary processes [7]. Eleven eyes, enucleated because of trauma but with no involvement of the iridocorneal angle, were analyzed. Seven eyes were treated for GL, while four of them had a normal IOP. Although the sample size was very small, a higher response, due to DA_1_ receptors, was noticed in eyes with normal IOP [7]. 

Mekki et al. demonstrated, in a double-blind study, that in 8 healthy volunteers without IOP alteration treated with oral bromocriptine (1.25 mg), IOP decreased (compared to placebo) at 3 and 4 hours after the administration, with no effects on systemic arterial pressure and on pupil's diameter [24, 25]. These results were later confirmed by a study involving patients suffering from Parkinson's disease and POAG. In fact, a decrease in IOP occurred when levodopa was replaced by bromocriptine per os (2.5 mg) [26]. This drug has effects on the production of prolactin, and Mekki et al. showed that the eye drops containing bromocriptine can reduce IOP compared to placebo [24, 25]. 

In a double-blind, randomized, and prospective study, IOP was measured in 24 healthy subjects at baseline and 2, 4, and 6 hours after the instillation of topical timolol (0.25%) and bromocriptine (0.05%). Both bromocriptine and timolol decreased IOP significantly from baseline with no difference between them at 2 and 4 h, but, at 6 h, bromocriptine was more effective than timolol [27]. 

Al-Sereiti et al. showed that the hypotensive effect of pergolide (25 micrograms orally) was antagonized by a pretreatment with oral domperidone (15 mg), a DA_2_ receptor low-selective peripheral antagonist [28]. 

DA_1_ receptor agonists were topically administered (DA, ibopamine, fenoldopam, and 3B90) in a study performed on 20 patients with POAG [29]. The authors demonstrated that the increase of IOP after the administration of those molecules was not inhibited neither by the administration of haloperidol nor by already known hypotensive drugs. Just the administration of a selective DA_1_ agonist (SCH-23390) was capable to reduce IOP, confirming that those receptors are involved in determining IOP.

Karnezis et al. showed that intravenous administration of angiotensin II, norepinephrine, and fenoldopam increased IOP in human subjects by the stimulation of DA_1_ receptors [30]. Subsequently, it was confirmed that the stimulation by fenoldopam could alter the outflow of the aqueous humor increasing IOP [31]. 

Among DA_1_ receptor agonists, we can also include the 3,4-di-isobutyrrylester of N-methyldopamine, better known as ibopamine [32, 33]. This widely studied molecule has the following pharmacological properties:

1. it increases the production of aqueous humor stimulating DA_1_ receptors both in healthy eyes and in glaucomatous ones;
2. it is a valid provocative test to study the efficiency of aqueous humor outflow pathways; in fact, in close relatives of patients with POAG, the instillation of ibopamine 2% causes an increase in IOP which would not occur in subjects without familiarity for POAG.

Those data explain that high IOP due to the administration of ibopamine is not caused by the increased production of aqueous humor but by an alteration of the outflow pathways [13, 32–34]. Ibopamine induces a cycloplegic mydriasis not acting on *α* receptors. Moreover, it can be used in hypotensive syndromes like anterior uveitis or damage of the ciliary body or after GL filtration surgery [33, 34].

Recently, Virno et al. [35] assessed the effect of ibopamine, a D1-dopamine agonist, on IOP in offsprings of parents with POAG as a consequence of outflow structures impairment. D1-dopaminergic stimulation due to ibopamine increases IOP as a result of increased production of the aqueous humor in participants with an impaired outflow. The study showed that after 2% ibopamine administration, there was a significant increase in aqueous humor production, both in glaucomatous and normal eyes. The intraocular hypertensive response due to ibopamine in normotensive eyes is a sign of initial outflow impairment and, therefore, a predisposition to intraocular hypertension and possible GL.

**Figure 2:**
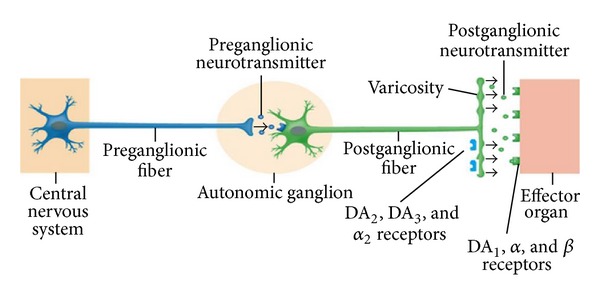
Prejunctional, indirect effects and postjunctional direct effects of dopamine (DA) and its analogues.

**Figure 3:**
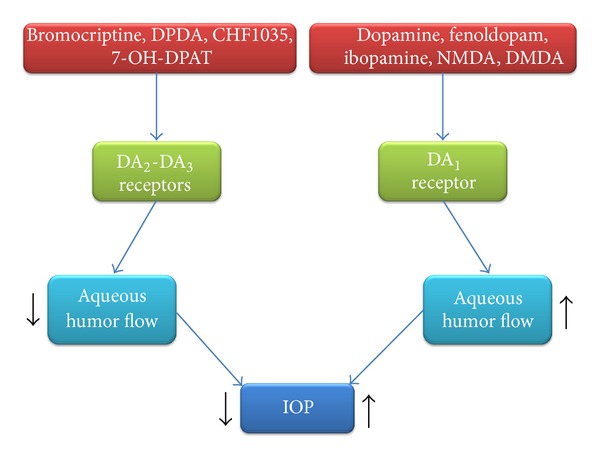
Effects of dopamine (DA) receptors on aqueous humor flow. DMDA: N,N-dimethyldopamine; DPDA: N,N-di-n-propyldopamine; NMDA: N-methyldopamine; 7-OH-DPAT: 7-hydroxy-2-dipropylaminotetralin.

After this overview, we can conclude that the effects of DA and its analogs on IOP are complex: they can have both direct effects (postjunctional) and indirect (prejunctional) ones. The postjunctional effects of DA agonists can stimulate *α*, *β*, and DA_1_ receptors. Similarly, indirect effects neuronal may be mediated by *α*_2_, DA_2_ and DA_3_ receptors (Figure 2). In particular, DA_1_ agonists seems to increase the production of aqueous humor with a consequent increase of IOP, while, DA_2_ agonists are responsible of a decrease of IOP (Figure 3). 

## 4. Conclusions 

Although several classes of drugs are known to reduce IOP, just 5 of them are used in clinical practice. It is still necessary to look for the most powerful (and safer) therapeutic agent in order to reduce the serious consequences of GL. From the studies carried out on animals and humans, discussed previously, we can conclude that DA_2_, and DA_3_ agonists have an important influence on the modulation of IOP, with significant implications for the management of patients affected by GL. 

Further studies are needed to clarify the role of the dopaminergic system and the usefulness of DA_2_ and DA_3_ agonists in lowering IOP.
